# Adult consequences of repeated nicotine and Δ^9^-tetrahydrocannabinol (THC) vapor inhalation in adolescent rats

**DOI:** 10.1007/s00213-024-06545-5

**Published:** 2024-01-29

**Authors:** Arnold Gutierrez, Kevin M. Creehan, Yanabel Grant, Michael A. Taffe

**Affiliations:** Department of Psychiatry, University of California, 9500 Gilman Drive, San Diego, La Jolla, CA 92093 USA

**Keywords:** E-cigarette, Self-administration, Nicotine, Cannabis, Adolescent, Opioids

## Abstract

**Rationale:**

Use of electronic drug delivery systems (EDDS, “e-cigarettes”) to ingest nicotine and Δ^9^-tetrahydrocannabinol (THC) has surged in adolescents in the USA; five times as many high-school seniors vape nicotine daily using tobacco. At the same time, 19.5% of seniors use cannabis at least monthly, with 12% using EDDS to deliver it.

**Objectives:**

This study was conducted to examine the impact of repeated adolescent vapor inhalation of nicotine and THC in rats.

**Methods:**

Female Sprague–Dawley rats were exposed to 30-min sessions of vapor inhalation, twice daily, from post-natal day (PND) 31 to PND 40. Conditions included vapor from the propylene glycol (PG) vehicle, nicotine (60 mg/mL in the PG), THC (100 mg/mL in the PG), or the combination of nicotine (60 mg/mL) and THC (100 mg/mL). Rats were assessed on wheel activity, heroin anti-nociception and nicotine and heroin vapor volitional exposure during adulthood.

**Results:**

Nicotine-exposed rats exhibited few differences as adults, but were less sensitive to anti-nociceptive effects of heroin (1 mg/kg, s.c.). THC- and THC + nicotine–exposed rats were less spontaneously active, and obtained fewer nicotine vapor deliveries as adults. In contrast, THC-exposed rats obtained volitional heroin vapor at rates indistinguishable from the non-THC-exposed groups. Repeated THC exposure also caused tolerance to temperature-disrupting effects of THC (5 mg/kg, i.p.).

**Conclusions:**

These studies further confirm that the effects of repeated vapor exposure to THC in adolescence last into early to middle adulthood, including decreased volitional consumption of nicotine. Effects of repeated nicotine in adolescence were comparatively minor.

## Introduction

Popularity and widespread availability of electronic nicotine delivery systems (ENDS), most commonly referred to as e-cigarettes, is evidenced by survey data in the USA showing that 20–26% of high-school seniors vaped nicotine in the past month 2019–2021 (Miech et al. 2022). Daily nicotine vaping declined from 11.6% in 2019 to 5% in 2020–2021, but rates of daily cigarette smoking were 2–3% across 2019–2021 in these populations (ibid). There is an obvious concern that reductions in the harm associated with cigarette smoking will be at least partially replaced by similar and/or novel harms associated with vaping for nicotine content. Concerningly, pre-teen rates of nicotine use went up during the initial months of COVID-19–related stay-at-home orders, while alcohol use declined (Pelham et al. 2021). A recent review points out the need for scientific research on the health impact of ENDS-based exposure to nicotine and furthermore identifies several domains in which adolescents have previously been found to be at increased risk (Fowler et al. 2018). In parallel with the use of cigarettes and ENDS to ingest nicotine, significant numbers of human adolescents exhibit high rates of cannabis use. Approximately 6% of high-school seniors report using cannabis daily/near daily, and 20–23% endorsed past-month use, from 1997 to 2021; with 12% reporting that they vaped cannabis in the past month in 2020–2021 (Johnston et al. 2021; Miech et al. 2022). Some individuals use ENDS devices for ingesting opioids (Blundell et al. 2018); however, there is a lack of specific study on these practices (Morris et al. 2023). Thus, these systems are more accurately termed electronic drug delivery systems (EDDS).

Human adolescents and young adults who initiate cannabis use are at increased risk for later nicotine use, and vice versa (Romm et al. 2023; Taylor et al. 2017; Temourian et al. 2023); however, multiple trajectories of cannabis and nicotine co-use have been reported (Rabinowitz et al. 2023), including simultaneous consumption (Hinds et al. 2023). This picture is further complicated by race and ethnicity, e.g., a study found African-American youth had reduced prevalence of nicotine vaping and nicotine/cannabis co-vaping, but increased prevalence of cannabis vaping, relative to white youth (Liu et al. 2023). Similar racial differences in preference for nicotine versus cannabis are also observed in adults (Boyle et al. 2021). In a twin study, women who used cannabis had increased odds of smoking and nicotine dependence (Agrawal et al. 2008). Additional work shows that adolescents who co-use nicotine and cannabis are at increased risk for higher use of cannabis and nicotine in young adulthood (Dunbar et al. 2020; Tucker et al. 2019). Relatedly, the children of mothers who use cannabis regularly are more likely to be nicotine dependent by age 22 (De Genna et al. 2022). Given this diversity and complex interrelatedness of cannabis and nicotine use, it is necessary to use controlled animal models to clearly establish the neuropharmacological risks of cannabis and/or nicotine exposure in adolescence apart from the influence of numerous human psychosocial factors. The goal of this study was therefore to determine any lasting impact of repeated vapor inhalation of nicotine and THC in adolescent rats on the effects of nicotine in adulthood.

For this, we used an EDDS-based system that has been previously shown effective in delivering nicotine to adult rats (Javadi-Paydar et al. 2019b; Lallai et al. 2021; Montanari et al. 2020), adolescent rats (Gutierrez et al. 2022), and to rat pups in utero (Breit et al. 2022; Hussain et al. 2022). EDDS-based systems have shown efficacy for nicotine self-administration in rats (Lallai et al. 2021; Smith et al. 2020) and mice (Cooper et al. 2021; Henderson and Cooper 2021), and have been used to show that repeated daily exposure to nicotine vapor leads to significant withdrawal following discontinuation in adult rats (Montanari et al. 2020). We have shown that repeated exposure to the major psychoactive constituent of cannabis, Δ^9^-tetrahydrocannabinol (THC), via vapor inhalation during adolescence in rats produces lasting consequences in adulthood. This included alterations in hypothermic and anti-nociceptive effects of THC in both sexes, increased food consumption in male rats and increased self-administration of fentanyl in female rats (Nguyen et al. 2020b). Similarly, twice daily vapor exposure to nicotine from PND 31 to 40 led to lasting alterations in the self-administration of nicotine by vapor inhalation in female, but not male, rats (Gutierrez et al. 2022). Thus, the overall approach of twice-daily adolescent vapor inhalation in brief sessions (30 min) using female rats was selected for this investigation.

This study exposed groups of adolescent female rats to twice daily inhalation of vapor from the propylene glycol (PG) vehicle, including nicotine (60 mg/mL in the PG), THC (100 mg/mL in the PG) or the combination of nicotine (60 mg/mL) and THC (100 mg/mL) for 10 days to determine lasting effects in adulthood. Rats were evaluated in adulthood for thermoregulatory and anti-nociceptive responses to THC and heroin, spontaneous locomotor activity on a wheel, the effect of nicotine on wheel activity, as well as on volitional nicotine and heroin exposure.

## Methods

### Subjects

Female Sprague–Dawley (Envigo/Harlan) rats (*N* = 32) were used for this study. The vivarium was kept on a 12:12 h reversed light–dark cycle, and behavior studies were conducted during the vivarium dark period. Food and water were provided ad libitum in the home cage. Animal body weights were recorded weekly, beginning at 6 weeks of age (PND 36) and continuing through the end of the study. Experimental procedures were conducted in accordance with protocols approved by the Institutional Animal Care and Use Committee of the University of California, San Diego, and consistent with recommendations in the NIH Guide (Garber et al. 2011).

### Drugs

Nicotine bitartrate (Sigma Pharmaceuticals LLC; North Liberty, IA) or heroin HCl (NIDA Drug Supply) were dissolved in propylene glycol (PG) for vapor inhalation studies and dissolved in physiological saline for subcutaneous injection studies. Concentrations of 30 and 60 mg/mL in the propylene glycol (PG) vehicle were used for nicotine and 50 mg/mL for heroin. THC (100 mg/mL) was suspended in PG at a concentration of 100 mg/mL for vapor inhalation studies and suspended in a 1:1:18 (ethanol:cremophor:saline) vehicle for intraperitoneal injection studies. PG was used as the vapor vehicle for consistency and comparability with our prior reports on the impact of nicotine, THC and heroin vapor inhalation (Gutierrez et al. 2021; Javadi-Paydar et al. 2019a, b; Taffe et al. 2021a).

### Apparatus

#### Vapor inhalation

An e-cigarette–based vapor inhalation system (La Jolla Alcohol Research, Inc.) which has previously been shown to deliver active doses of THC, cannabidiol, heroin, oxycodone, methamphetamine, and nicotine (Gutierrez et al. 2021; Javadi-Paydar et al. 2019a, b; Nguyen et al. 2016a, 2019) was used for these studies. Vapor was delivered into sealed vapor exposure chambers (152 mm W × 178 mm H × 330 mm L; La Jolla Alcohol Research, Inc., La Jolla, CA, USA) through the use of e-vape controllers (Model SSV-3 or SVS-200; 58 watts, 0.24–0.26 ohms, 3.95–4.3 V, ~ 214 °F; La Jolla Alcohol Research, Inc., La Jolla, CA, USA) to trigger SMOK Baby Beast Brother TFV8 sub-ohm tanks. Tanks were equipped with V8 X-Baby M2 0.25 Ω coils which were replaced approximately halfway through the repeated-exposure experiment and, in general, any time when a decrement in the vapor cloud was observed. MedPC IV software was used to schedule and trigger vapor delivery (Med Associates, St. Albans, VT, USA). The apparatus and settings were the same for all drug conditions. The chamber air was vacuum-controlled by a chamber exhaust valve (i.e., a “pull” system) to flow room ambient air through an intake valve at ~ 1 L per minute. This also functioned to ensure that vapor entered the chamber on each device triggering event. The vapor stream was integrated with the ambient air stream once triggered. Airflow was initiated 30 s prior to, and discontinued 10 s after the initiation of, each puff. Vapor was visibly cleared from the chambers after the 30-s airflow prior to subsequent puffs. Each vapor puff delivery totaled 6 s in duration, and there were a total of six puffs delivered (i.e., at 0, 5, 10, 15, 20, and 25 min).

### Activity wheels

Experimental sessions were conducted in white illuminated procedure rooms with activity wheels that attached to a typical housing chamber with a door cut to provide access to the wheel (Med Associates; Model ENV-046), using approaches previously described (Gilpin et al. 2011; Miller et al. 2013; Taffe et al. 2021b). Rats were given access to the wheel in acute 30-min sessions during which wheel rotation (quarter-rotation resolution) activity was recorded at 5- or 10-min intervals. One habituation session was conducted for each animal prior to initiating the following experimental sessions.

### Experiments

#### Experiment 1: Effect of repeated adolescent nicotine and THC inhalation on nociception and body temperature

A cohort of 32 female Sprague–Dawley rats arrived in the laboratory at PND25, were randomly assigned to groups (*N* = 8 per group) by cage (*N* = 2 per cage) and were exposed to 30-min vapor sessions in white light (during the vivarium dark cycle) twice per day (6 h apart; 800/1400 h) for 10 consecutive days from PND 36 to 45. Vapor conditions included PG, nicotine (60 mg/mL), THC (100 mg/mL) or the combination of nicotine (60 mg/mL), and THC (100 mg/mL) tested in separate groups. The higher concentration of nicotine compared with a prior experiment (Gutierrez et al. 2024) was selected to match the concentration used in JUUL devices and to provide some dose–effect information relative to that prior study. On PND 71, the groups were injected with THC (0.725 mg/kg, i.p.) and assessed on tail withdrawal and rectal temperature before and 30 min after injection. On PND 80–81, groups were injected with THC (5.0 mg/kg, i.p.) and assessed on tail withdrawal and rectal temperature before, and 30, 60, and 120 min after injection.

#### Experiment 2: Effect of repeated adolescent nicotine and THC inhalation on wheel activity

From PND 114 onward, the groups of female rats from Experiment 1 were assessed for wheel activity in 30-min sessions in the dark. Animals first received a habituation session on 1 day and then a baseline session 3 days later, with no prior treatment, to assess any lasting group differences associated with the adolescent vapor treatment conditions. Thereafter the effects of 30-min inhalation of PG or nicotine (30 mg/mL; in the dark) were assessed with the order counterbalanced within the groups and a 6-day interval between assessments. Wheel activity without any drug exposure was re-determined PND 150–151.

#### Experiment 3: Effect of repeated adolescent nicotine and THC inhalation on volitional nicotine vapor consumption

The rats were assessed for volitional exposure to nicotine (30 mg/mL) vapor in 30-min sessions in the dark from PND 155 or 156 onward. Four chambers with nose-poke manipulanda and two with lever manipulanda were used with the assignment of lever vs. nose-poke boxes counterbalanced across treatment groups and day cohorts, see below. A response on the drug-associated manipulandum (fixed ratio 1 response requirement) resulted in illumination of the cue light and delivery of a 1-s puff of vapor. This was followed by a 20-s timeout during which the cue light remained illuminated and hole/lever responses were recorded, but led to no consequences. Sessions were scheduled no more frequently than every other day since prior work has indicated that sequential days of intravenous nicotine self-administration can produce declining trends (O’Dell and Koob 2007). Cohorts consisting of half of each of the treatment groups were run on alternating days. A facilities flooding emergency occurred around the tenth (second cohort) and eleventh (first cohort) acquisition sessions and scheduled remediation and repairs disrupted access to testing rooms thereafter. As there may have been effects of the developing water leak in the building, and staff response to it, analysis of the acquisition phase was limited to the initial 9 sessions. Animals were idled for 3 weeks and then re-started under the FR1, termed session 10 here for convenience. After session 12, the schedule of reinforcement was increased to FR5 for three sessions and then restored to FR1 for three additional sessions. Menthol has been reported to enhance mouse vapor self-administration of nicotine, thus nicotine 30 mg/mL with 5% menthol was assessed for 3 sessions, followed by nicotine 60 mg/mL with 5% menthol for an additional 8 sessions. Animals were switched to different operant boxes (with the manipulandum unchanged for each rat) during the last six of these eight sessions, to determine if individual differences were confounded with the specific operant box.

#### Experiment 4: Effect of heroin injection on nociception and body temperature

The impact of heroin (0.0, 0.56, 1.0, 1.56 mg/kg, s.c.) on tail withdrawal and rectal temperature was assessed from 42 to 43 weeks of age (~ PND 296–306) in a counterbalanced order. The approach was as described for Experiment 1.

#### Experiment 5: Effect of repeated adolescent nicotine and THC inhalation on volitional heroin vapor exposure

Access to volitional vapor was restarted after Experiment 4, at approximately 46–49 weeks of age (~ PND 324–345). Animals were assessed in 30-min sessions, in the dark, with the opportunity to obtain puffs of heroin (50 mg/mL) vapor under a FR1 schedule of reinforcement for four sessions. These sessions were compared with the final prior nicotine (60 mg/mL + menthol) sessions in the analysis. Tail withdrawal was evaluated before and after the first heroin session.

### Data analysis

Data were analyzed by analysis of variance, save that mixed-effect models were used in any cases of missing values, including when percent of responses on the drug-associated manipulandum were undefined due to no drug-associated responses being emitted. Within-subjects factor of time (after vapor initiation/injection or wheel access), acute treatment condition or self-administration session, and a between-subjects factor for adolescent treatment group were included as relevant. Due to a main effect of receiving THC during adolescence in the study of rectal temperature after THC administration, subsequent analysis of data for these groups included, *a priori*, an initial four-group assessment using a three factor (time/session/acute treatment, presence/absence of THC, presence/absence of nicotine) analysis, followed by examining the data in a two factor analysis collapsed across the two groups that received/did not receive THC and then a two factor analysis collapsed across the two groups that received/did not receive nicotine (see Table 1), with follow up post hoc exploration of significant main effects or interactions. In all analyses, a criterion of *P* < 0.05 in two-sided analysis was used to infer that a significant difference existed. Any significant main effects were followed with post hoc analysis using Tukey (multi-level factors), Dunnett (comparison with a control condition), or Sidak (two-level factors) correction. All statistical analyses used Prism for Windows (v. 9.5.1–10.0.0; GraphPad Software, Inc., San Diego, CA).

**Table 1:** Factorial design for the primary analysis

	No-THC	THC
No nicotine	PG	THC 100 mg/mL
Nicotine	Nicotine 60 mg/mL	THC + nicotine

## Results

### Repeated THC and nicotine vapor exposure

There were no group differences in body weight associated with the adolescent inhalation treatment across the study interval. *N* = 30 completed all scheduled studies and were euthanized between PND 437 and 444 (~ 62–63 weeks of age). One rat (PG group) was observed to develop a clinically concerning solid mass and was euthanized PND 319, and one rat (PG group) was found dead of unknown causes PND 398.

#### Experiment 1: Effect of repeated adolescent nicotine and THC inhalation on nociception and body temperature

**Fig. 1:**
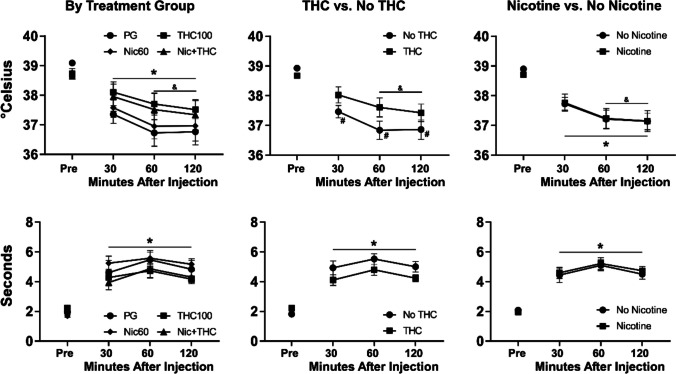
Mean (± SEM) rectal temperature and tail-withdrawal latencies following injection with THC (5.0 mg/kg, i.p.) are depicted for all four treatment groups, for groups which did or did not receive THC and for groups which did or did not receive nicotine. A significant difference from the pre-injection value, collapsed across group, is indicated with *. A significant difference from the pre-injection value, within each group, is indicated with #. A significant difference from the 30-min value, collapsed across group, is indicated with &

Injection of 0.725-mg/kg THC, i.p., produced threshold effects (not shown); there was a significant effect of time (before/after THC) for both rectal temperature [F (1, 28) = 24.46; *P* < 0.0001] and tail withdrawal latency [F (1, 28) = 36.30; *P* < 0.0001], but no significant group differences were confirmed in the initial study. The injection of 5 mg/kg THC, i.p., significantly reduced rectal temperature and increased tail withdrawal latencies as is shown in Fig. 1. The three factor ANOVA confirmed a significant effect of time [F (1.825, 51.10) = 38.60; *P* < 0.0001] and the interaction of time with adolescent treatment group [F (3, 84) = 3.37; *P* < 0.05] on rectal temperature. The follow up two-factor ANOVA collapsed across the nicotine exposure factor likewise confirmed a significant effect of time [F (3, 90) = 40.45; *P* < 0.0001] and the interaction of time with THC/no-THC group [F (3, 90) = 3.54; *P* < 0.05] on rectal temperature. The Tukey post hoc test further confirmed that temperature was significantly lower than the pre-injection temperature 30, 60, and 120 min after injection, within each of the adolescent THC/no-THC groups. The post hoc test did not, however, confirm any significant differences between the groups at any of the time points.

The three-way analysis of the tail withdrawal latencies confirmed a significant effect of time [F (2.495, 69.87) = 52.71; *P* < 0.0001], but not of adolescent treatment group by itself or in interaction with the time factor. The post hoc analysis confirmed that withdrawal latency was slower than the baseline value at every post-injection timepoint. The follow up two-factor analyses using the nicotine/no-nicotine groupings did not confirm any significant effects of group on temperature or tail-withdrawal latency.

#### Experiment 2: Effect of repeated adolescent nicotine and THC inhalation on wheel activity

##### Baseline test for wheel activity, PND 114

**Fig. 2:**
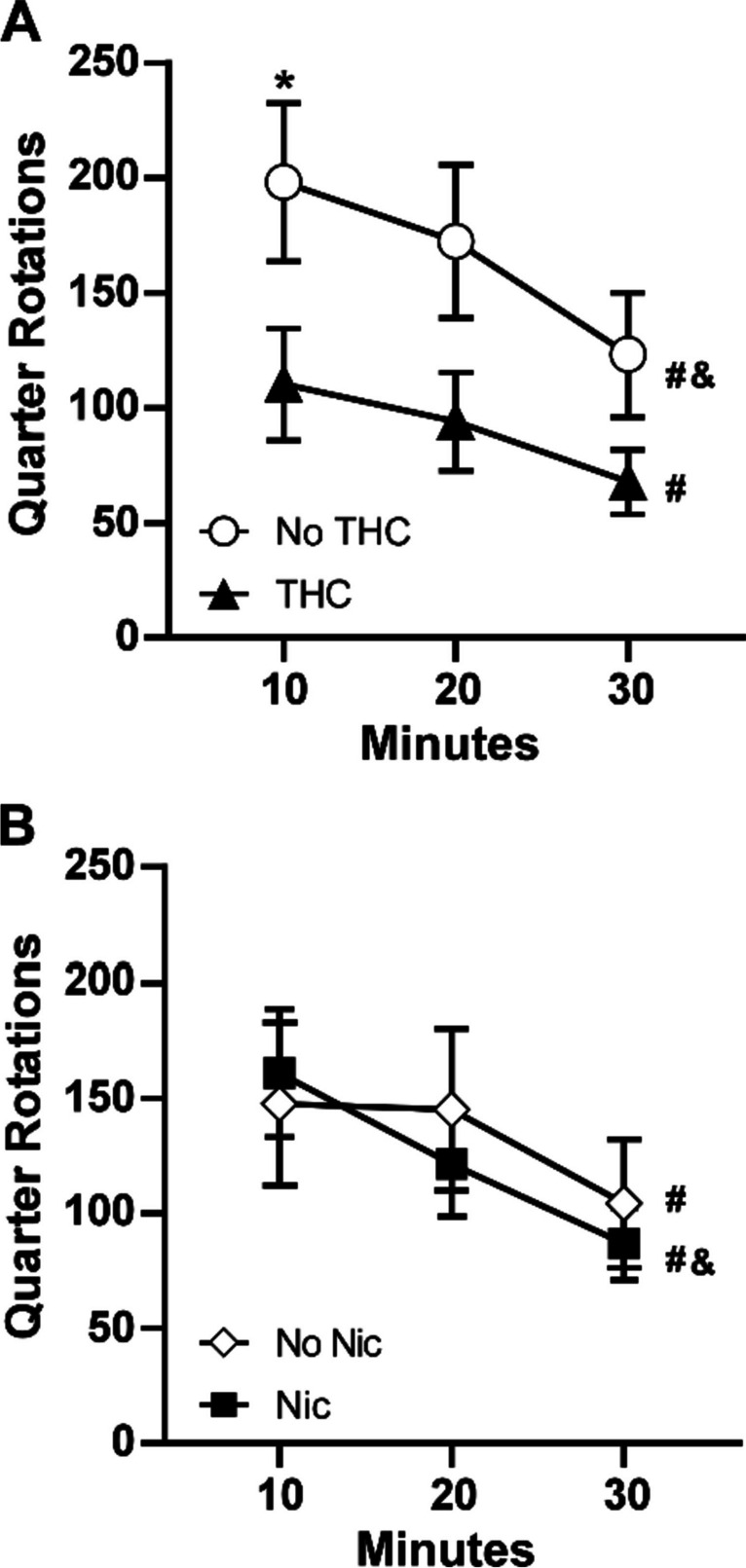
Mean (*N* = 16; ± SEM) quarter rotations of the wheel for the groups that received THC or no-THC (**A**) or that received nicotine or no nicotine (**B**). A significant difference between groups is indicated with *, a difference from the 10-min bin with # and a difference from the 20-min bin with &

The baseline (i.e., without any acute drug exposure) wheel activity assessed in early adulthood (PND 114) differed between the two groups that received adolescent vapor exposure to THC (alone or in combination with nicotine) and the two that did not (PG and nicotine only), as depicted in Fig. 2. The initial three-factor analysis confirmed that there was an effect of time [F (1.719, 48.14) = 11.51; *P* < 0.0005], and of the presence/absence of THC [F (1, 28) = 4.36; *P* < 0.05], but not of the presence/absence of nicotine, in the adolescent vapor on baseline wheel activity. Post hoc exploration of the follow-up analysis of the rats grouped by THC/no-THC [time: F (1.689, 50.67) = 11.77; *P* = 0.0001; group: F (1, 30) = 4.46; *P* < 0.05; interaction: n.s.] or by nicotine/no nicotine [time: F (1.737, 52.10) = 11.92; *P* = 0.0001; group: n.s.; interaction: n.s.] further confirmed significantly less wheel activity in the first 10 min for the THC-exposed groups.

##### Impact of nicotine on wheel activity

**Fig. 3:**
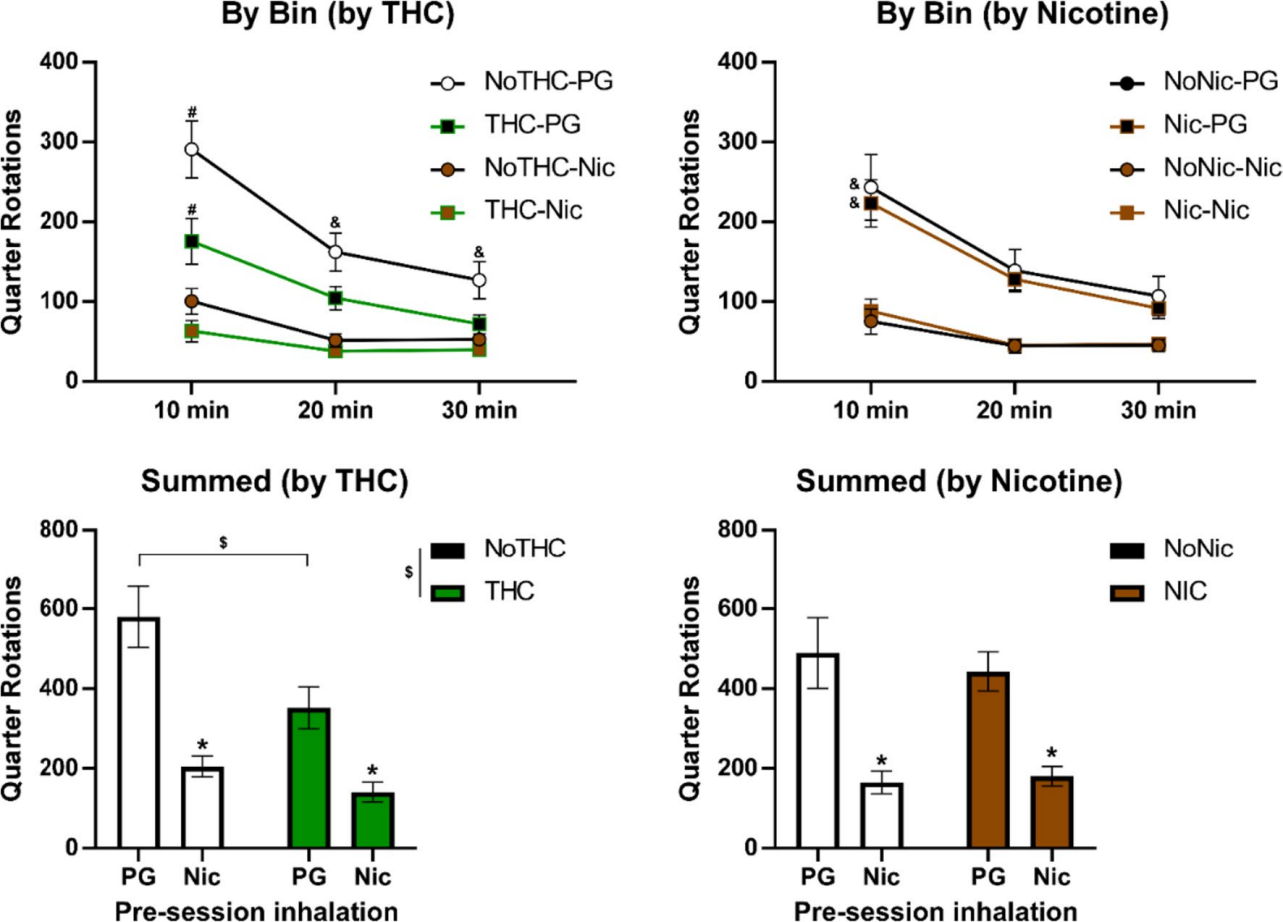
Mean (± SEM) wheel activity following inhalation of nicotine (30 mg/mL) vapor. A significant difference from all other groups/conditions is indicated by # and a difference from each nicotine vapor condition by &. A significant difference between adolescent treatment groups is indicated with $ and significant difference associated with pre-session inhalation condition with *

Nicotine vapor inhalation before the session significantly suppressed wheel activity (Fig. 3), as was confirmed by a significant effect of acute treatment condition [F (1.000, 30.00) = 51.99; *P* < 0.0001] in the three factor analysis of the THC vs. no-THC groups wheel activity by 10-min time bin. There were also significant effects of THC/no-THC group [F (1, 30) = 6.44; *P* < 0.05], of time [F (1.797, 53.90) = 59.60; *P* < 0.0001], as well as of the interactions of Time with THC/No-THC group [F (2, 60) = 4.21; *P* < 0.05] and with the acute treatment condition [F (1.303, 39.10) = 27.73; *P* < 0.0001]. The three-factor analysis of the nicotine vs. no-nicotine groups confirmed significant effects of time [F (1.816, 54.49) = 52.30; *P* < 0.0001], of pre-treatment condition [F (1.000, 30.00) = 46.58; *P* < 0.0001], and the interaction of time with pre-treatment [F (1.000, 30.00) = 46.58; *P* < 0.0001], but there were no significant effects of adolescent treatment group.

Analysis of total session wheel activity with a three factor ANOVA confirmed a significant effect of acute treatment condition [F (1, 28) = 53.11; *P* < 0.0001], and of the adolescent THC [F (1, 28) = 6.32; *P* < 0.05]; however, there was no main effect of adolescent nicotine, nor any interactions with nicotine confirmed. The follow-up two factor ANOVA of the THC/no-THC grouping again confirmed main effects of adolescent treatment [F (1, 30) = 6.44; *P* < 0.05] and of acute treatment condition [F (1, 30) = 51.99; *P* < 0.0001], but there was no significant interaction [F (1, 30) = 4.06; *P* = 0.0529] of factors.

The post hoc test confirmed significant suppressive effects of acute nicotine within each of the THC/no-THC adolescent treatment groupings and a significant difference between adolescent treatment groupings for PG treatment only. The follow-up two-factor ANOVA of the nicotine/no-nicotine grouping again confirmed main effects of pre-treatment condition [F (1, 30) = 51.99; *P* < 0.0001], but there were no significant effects of nicotine/no-nicotine adolescent treatment grouping or of the interaction of factors. The post hoc test again confirmed significant suppressive effects of acute nicotine within each of the nicotine/no-nicotine adolescent treatment groupings.

##### Baseline test for wheel activity, PND 150–151

**Fig. 4:**
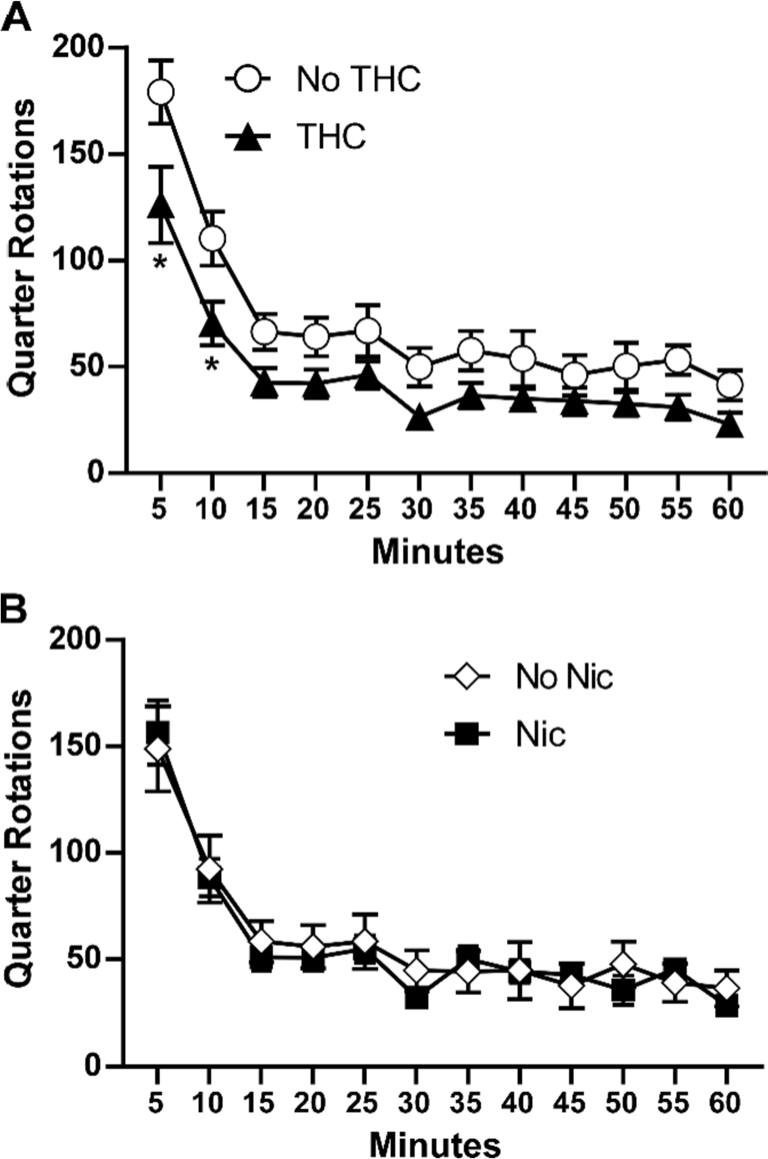
Baseline wheel activity PND 150–151. Mean (*N* = 16; ± SEM) quarter rotations of the wheel for the groupings that received THC or no THC (**A**) or that received nicotine or no nicotine (**B**). A significant difference between groups is indicated with *

When assessed on PND 150–151, the animals exposed to THC (alone or in combination with nicotine) still emitted less wheel activity than those without THC exposure (PG and Nic alone), as depicted in Fig. 4. In a three-factor ANOVA, there were significant effects of time [F (11, 308) = 70.97; *P* < 0.0001], of THC condition [F (1, 28) = 5.09; *P* < 0.05] and of the interaction of time with THC condition [F (11, 308) = 1.97; *P* < 0.05]. The post hoc exploration of the THC/no-THC groupings further confirmed a significant difference between the groups for the 5- and 10-min bins. No significant effects of nicotine condition alone or in interaction were confirmed.

#### Experiment 3: Effect of repeated adolescent nicotine and THC inhalation on volitional nicotine vapor exposure

**Fig. 5:**
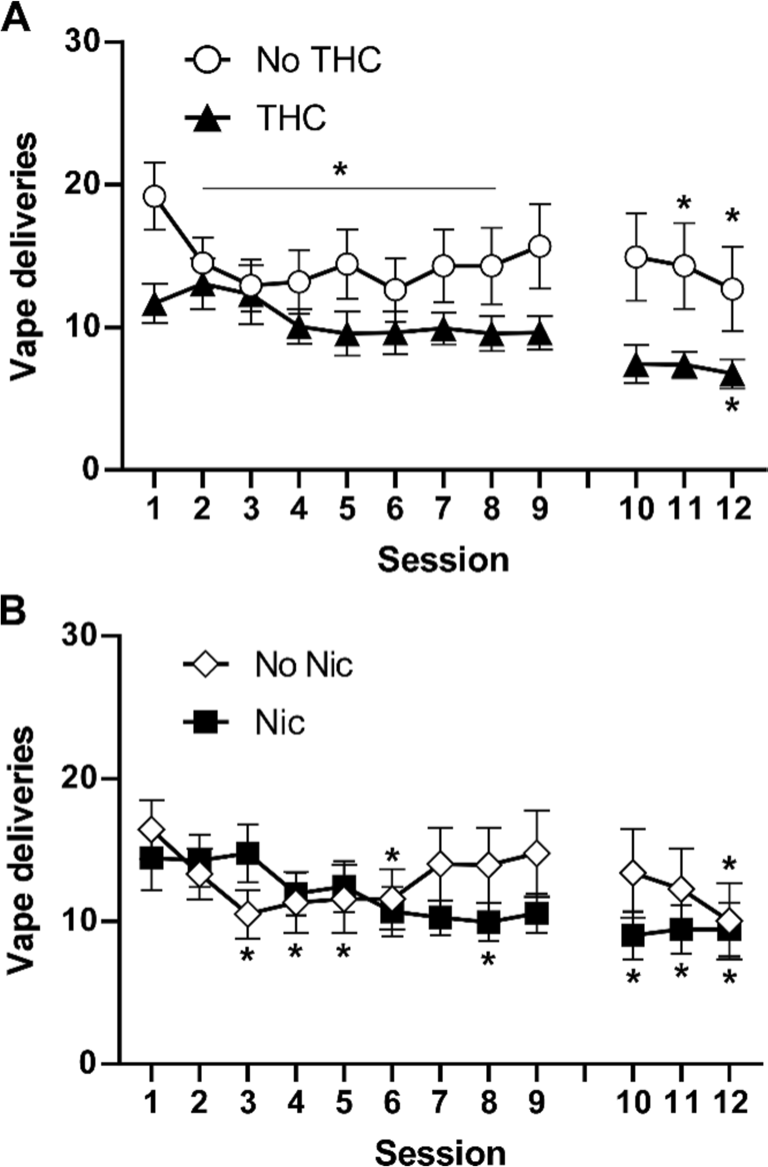
Acquisition of nicotine vapor (30 mg/mL) self-administration. Mean (N = 16; ± SEM) vapor deliveries obtained during acquisition for the groupings that received THC or no THC (**A**) or that received nicotine or no nicotine (**B**). A significant difference from the first session within group is indicated with *. Animals were idled for 3 weeks between sessions 9 and 10 due to an unavoidable facilities interruption

The rats that were exposed to THC (alone or in combination with nicotine) obtained fewer vapor.

deliveries than those without THC exposure (PG and Nic alone) by the end of acquisition (Fig. 5). The unavoidable interruption after session 9 due to facilities emergency was followed by a return to approximately the same number of vapor deliveries in sessions 10–12. The initial three-factor ANOVA confirmed significant effects of session [F (8, 224) = 2.86; *P* = 0.005] and the interaction of session with the presence/absence of THC [F (8, 224) = 1.98; *P* < 0.05], of the interaction of session with the presence/absence of nicotine [F (8, 224) = 3.42; *P* < 0.005], and the interaction of all three factors [F (8, 224) = 2.16; *P* < 0.05] on vapor deliveries. The follow-up analysis of the impact of THC confirmed significant effects of session [F (11, 330) = 3.26; *P* < 0.0005] and the interaction of adolescent vapor condition with session [F (11, 330) = 1.94; *P* < 0.05], as did the analysis for the impact of nicotine [session: F (11, 330) = 3.34; *P* < 0.0005; interaction: F (11, 330) = 2.76; *P* < 0.005]. The follow-up Dunnett post hoc analysis of the impact of adolescent THC confirmed that compared with the first session, fewer deliveries were obtained by the no-THC group on sessions 2–8, 11–12 and by the THC group on sessions 12. The follow-up Dunnett post hoc analysis of the impact of adolescent nicotine confirmed that compared with the first session, fewer deliveries were obtained by the no-nicotine group on sessions 3–6, 12 and by the nicotine group on sessions 8, 10–12.

**Fig. 6:**
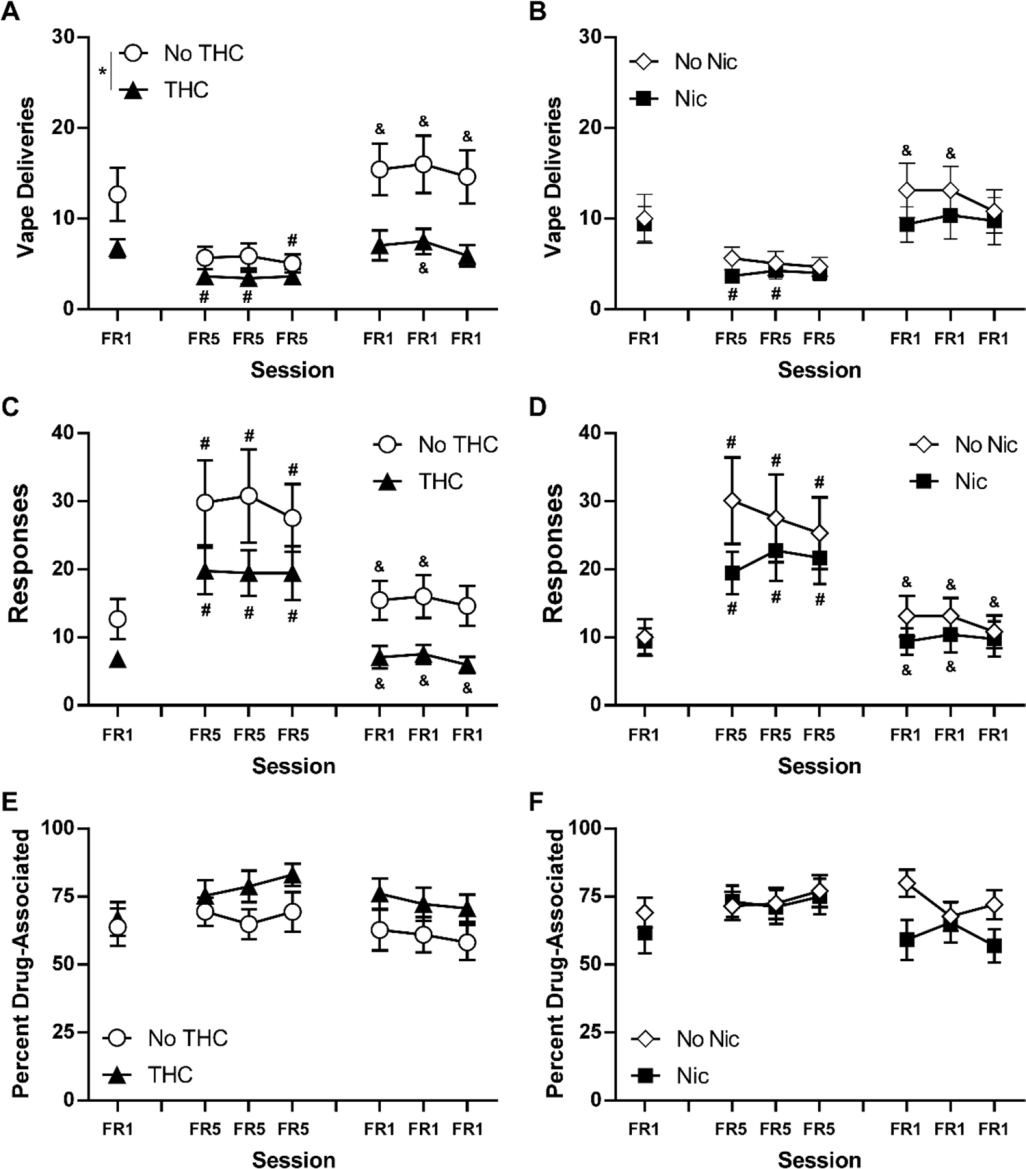
Nicotine vapor (30 mg/mL) self-administration. Mean (*N* = 16; ± SEM) vapor deliveries (**A**, **B**), total responses on the drug-associated manipulandum (**C**, **D**) and percent of responses directed to the drug-associated manipulandum (**E**, **F**) for the groupings that received THC or no THC (**A**, **C**, **E**) or that received nicotine or no nicotine (**B**, **D**, **F**). A significant difference between groups is indicated with *. Within group, significant differences from the initial FR1 session are indicated with # and from the third FR5 session with &

In the FR 5 experiment, the THC-exposed rats obtained fewer vapor deliveries (Fig. 6A), and the three factor ANOVA confirmed a significant effect of THC/no-THC condition [F (1, 28) = 5.90; *P* < 0.05], of the FR condition [F (6, 168) = 16.90; *P* < 0.0001], and of the interaction of THC with FR condition [F (6, 168) = 4.02; *P* = 0.001] on vapor deliveries. There were no significant effects of the adolescent nicotine factor alone or in interaction with other factors. Post hoc analysis of the FR factor after the follow up two-factor ANOVA, collapsed across all groups, confirmed that significantly fewer vapor deliveries were obtained in all three FR5 sessions compared with the FR1 baseline and compared with all three post-FR5 FR1 sessions.

In the follow-up analysis of the THC factor, the Tukey post hoc analysis further confirmed that within the no-THC group fewer vape deliveries were obtained on the third FR5 session compared with the FR1 baseline. In addition, significantly fewer vape deliveries were obtained in all three FR5 sessions compared with all three of the following FR1 sessions. Intakes also differed significantly between the second and third FR5 sessions. Within the THC group, significantly fewer vape deliveries were obtained in the first and second FR5 sessions compared with the FR1 baseline. In addition, significantly more vape deliveries were obtained in the second FR1 session after the FR5 sessions compared with all three FR5 sessions.

Analysis of the total drug–associated responses with a three-factor ANOVA confirmed a significant effect of FR condition [F (6, 168) = 21.94; *P* < 0.0001] but not of either adolescent treatment factor (Fig. 6C). Post hoc analysis of the FR factor after the follow up two-factor ANOVA collapsed across all groups, confirmed that significantly more responses were emitted in all three FR5 sessions compared with the FR1 baseline and compared with all three post-FR5 FR1 sessions.

Analysis of the percent of responses directed at the drug-associated manipulandum (Fig. 6E) with a three-factor mixed-effects analysis confirmed a significant effect of THC condition [F (1, 28) = 5.16; *P* < 0.05] and of the interaction of THC with nicotine factor [F (1, 28) = 15.47; *P* < 0.0005]; no significant effects of FR condition were confirmed. The Tukey post hoc analysis comparing all four original groups confirmed that the nicotine exposed animals exhibited a significantly lower percentage of responses on the drug-associated manipulandum compared with the PG and combination groups.

Follow-up analysis of the adolescent nicotine versus no-nicotine groups did not confirm any significant effect of this factor on vape deliveries, total responses, or percent drug-associated responses. No detectable differences associated with the nose-poke vs. lever manipulanda were observed in the self-administration studies.

#### Experiment 4: Effect of heroin injection on nociception and body temperature

**Fig. 7:**
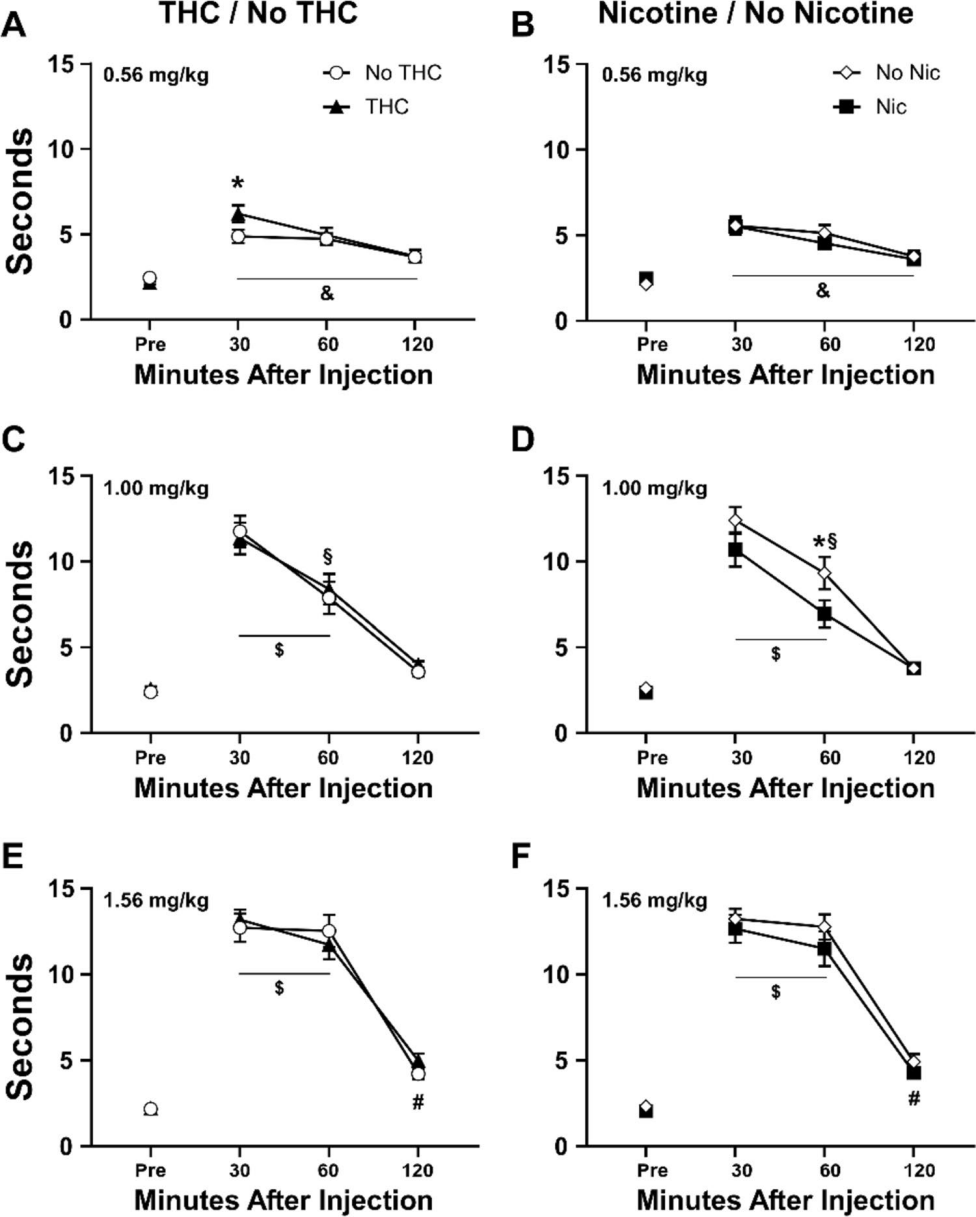
Mean (*N* = 16 per group; ± SEM) tail-withdrawal latencies following injection with heroin (0.56–1.56 mg/kg, s.c.) are depicted for groups which did or did not receive THC (**A**, **C**, **E**) and for groups which did or did not receive nicotine (**B**, **D**, **F**). A significant difference between groups is indicated with *. Across group, a significant difference from all other time points is indicated with &. A significant difference from the baseline is indicated with #. A significant difference from the baseline and 120 min is indicated with $

Heroin injection significantly slowed tail-withdrawal latency in a dose- and time-dependent manner (Fig. 7). The 3-factor analyses focused on the effect of time after injection for each dose. The analyses confirmed significant effects of time after vehicle [F (3, 56) = 10.89; *P* < 0.0001] (not shown; range 2.1–5.1 30–60 min post-injection), 0.56 mg/kg [F (3, 56) = 52.61; *P* < 0.0001], 1.0 mg/kg [F (3, 56) = 72.17; *P* < 0.0001], and 1.56 mg/kg [F (3, 56) = 178.1; *P* < 0.0001] heroin injection. There was a significant effect of the nicotine/no-nicotine factor for the 1.0 mg/kg dose [F (1, 56) =

6.64; *P* < 0.05] and an interaction of the THC/no-THC factor with time [F (3, 56) = 3.20; *P* < 0.05] after the 0.56 mg/kg heroin injection. Follow up post hoc analysis of the THC/no-THC groupings for the 0.56 mg/kg dose confirmed a significant difference 30 min after injection and the follow-up post hoc analysis of the nicotine/no-nicotine groupings for the 1.0 mg/kg dose confirmed a significant difference 60 min after injection.

There were no significant effects of heroin injection on body temperature confirmed in the analysis (not shown).

#### Experiment 5: Effect of repeated adolescent nicotine and THC inhalation on volitional heroin vapor exposure

**Fig. 8:**
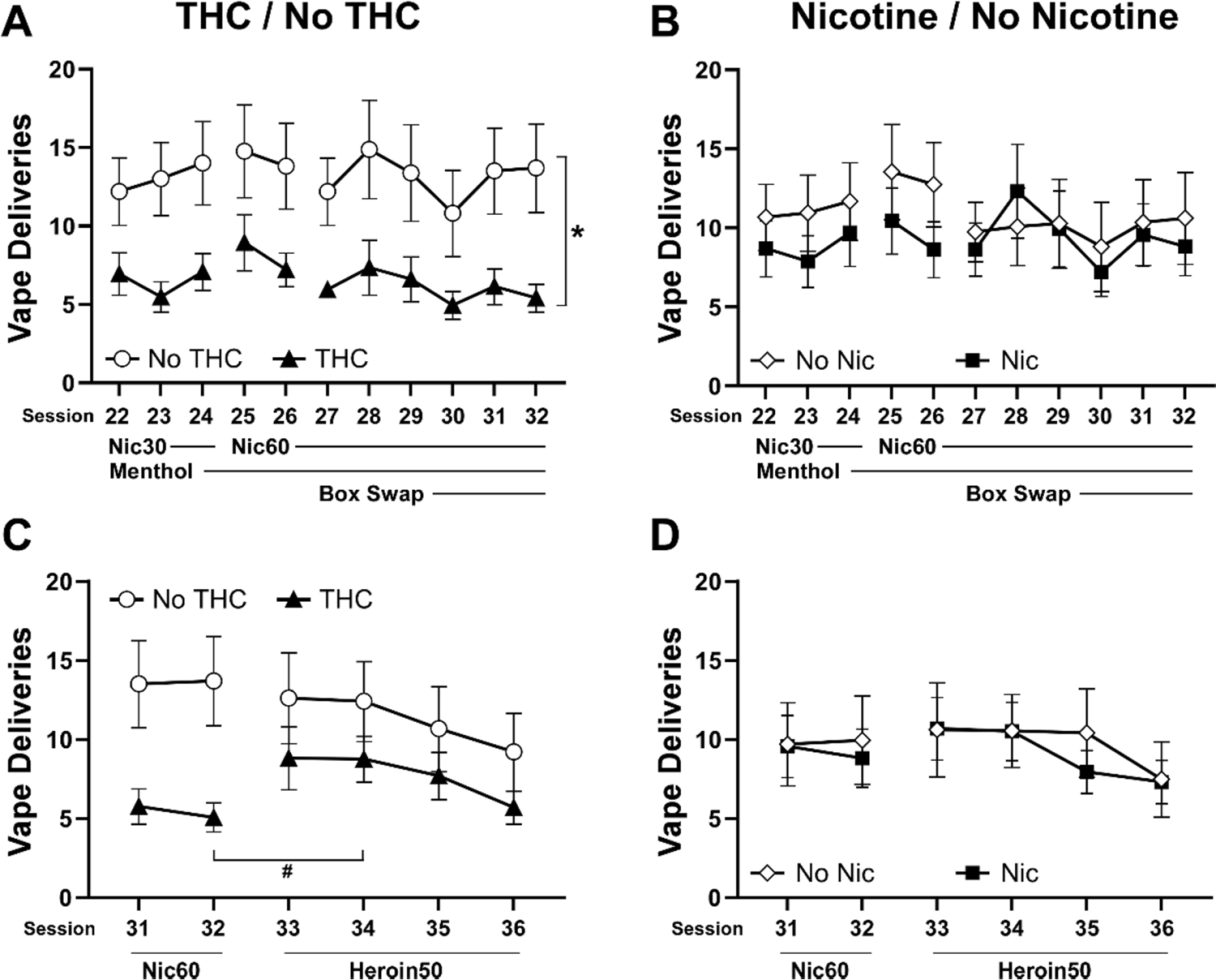
Mean (± SEM) vapor deliveries of nicotine (30, 60 mg/mL) and heroin (60 mg/mL) obtained by groups exposed as adolescents to PG, THC, nicotine or THC + nicotine vapor, grouped as the THC vs. no-THC groups, and as the nicotine versus no-nicotine groups. Sessions 31 and 32 are repeated in the lower panels for assessment of the change when heroin was introduced. A difference between sessions within group is indicated by # and a difference between groups, across sessions, is indicated with *

Responding for nicotine vapor under the FR1 response contingency after the break for the heroin anti-nociception study approximated the same levels (Fig. 8) as initially expressed at the end of the FR5/FR1 study (Fig. 6). Vape deliveries were unaffected by the addition of menthol, a change to the 60 mg/mL concentration and a change of operant box for each animal. The group differences remained consistent with the adolescent THC exposure groups obtaining fewer vapor deliveries than the groups that did not receive adolescent THC. The analysis confirmed a significant effect of THC/no-THC group [F (1, 29) = 6.11; *P* < 0.05] and of session [F (5.027, 145.8) = 2.39; *P* < 0.05], but the post hoc test did not further confirm any specific differences. No differences were observed between the nicotine and no-nicotine adolescent treatment groups.

The introduction of heroin (50 mg/mL) vapor as the reinforcer significantly increased vapor deliveries obtained by the THC-exposed groups. The analysis confirmed a significant interaction between group and session [F (5, 146) = 2.54; *P* < 0.05] and the post hoc Dunnett test confirmed that, relative to the final nicotine vapor session, the THC-exposed group obtained significantly more vapor deliveries on the second heroin session.

**Fig. 9:**
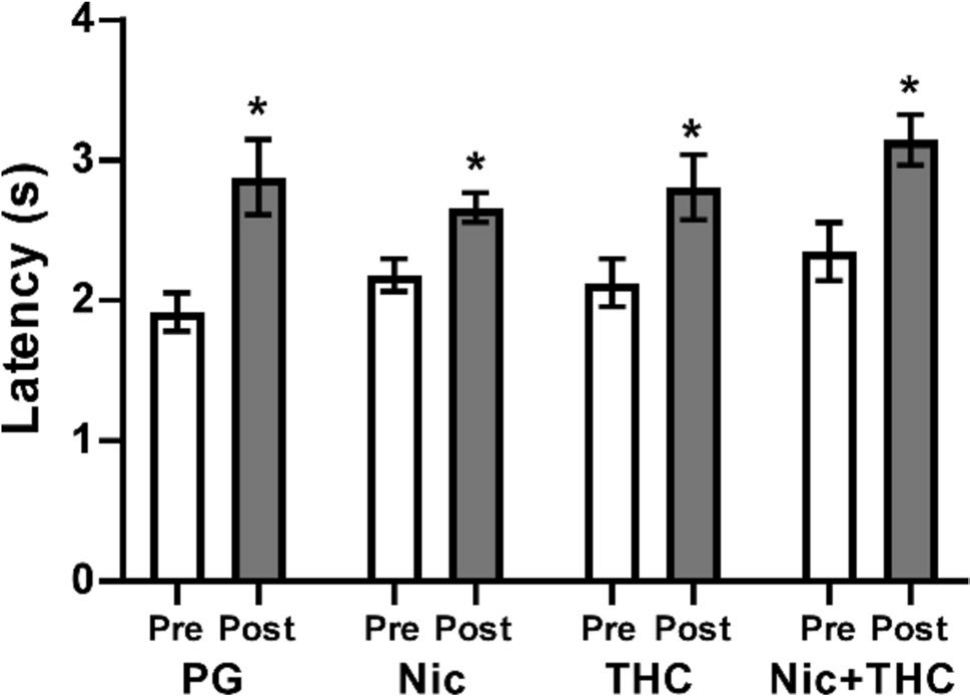
Mean (*N* = 8 per group; ± SEM) tail-withdrawal latencies before and after the first heroin vapor self-administration session are depicted for adolescent treatment groups. A significant difference between the pre- and post-session measurements is indicated with *

Nociception was assessed on the first day of heroin vapor availability. An anti-nociceptive effect was confirmed (pre/post: F (1, 27) = 79.45; *P* < 0.0001) and the post hoc test further confirmed that latencies were significantly slower after self-administration in all four adolescent treatment groups (Fig. 9). No differences between groups were confirmed.

## Discussion

The study demonstrates that there are physiological and behavioral effects of repeated adolescent exposure to Δ^9^-tetrahydrocannabinol (THC) via electronic drug delivery systems (EDDS) vapor inhalation that last into adulthood in female rats. Vapor exposure to THC produced a lasting tolerance to the temperature disrupting effects of THC, reduced spontaneous activity on an exercise wheel and decreased volitional responding for nicotine vapor. In contrast, there were no lasting differences in the baseline wheel activity levels or in the effects of acute nicotine pre-treatment associated with the adolescent exposure to nicotine vapor. This is unlike our prior result for adolescent females exposed to twice daily nicotine vapor inhalation PND 31–40 using the 30-mg/mL concentration (Gutierrez et al. 2022), which suggests the potential importance of dose to the outcome. The addition of nicotine to the THC during adolescent exposure did not significantly modify the impact of the THC.

### Wheel activity and thermoregulation

The rats exposed to nicotine (alone or in combination with THC) had statistically indistinguishable wheel activity compared with the rats which did not receive adolescent nicotine, in the baseline test as well as in both of the PG and nicotine inhalation conditions. In contrast, the rats that were exposed to THC (alone or in combination with nicotine) exhibited less baseline wheel activity as well as less activity under the PG challenge condition. The consistency of this pattern suggests it is a stable consequence of the repeated THC, and not an interaction of that treatment with the novelty of the wheel, e.g., as assessed on PND 114. The effect of nicotine, delivered by vapor inhalation, was to suppress wheel activity. This was similar to a suppressive effect of 0.4–0.8 mg/kg nicotine, s.c., injection on wheel activity previously reported (Bryson et al. 1981; Gutierrez et al. 2024). Twice daily adolescent vapor exposure to THC on 10 consecutive days (PND 36–45) produced lasting tolerance the thermoregulatory effects of THC in young adulthood, similar to a prior study with rats treated twice daily PND 35–39, 42–46 (Nguyen et al. 2020b). Similar thermoregulatory tolerance after twice daily repeated THC vapor exposure also has been reported for adult female rats (Nguyen et al. 2018, 2020a). This study extended those observations to show additional lasting behavioral consequences include reductions in spontaneous wheel activity.

As one minor caveat, one recent report suggest that vaping nicotine and THC together leads to lower plasma drug levels than when the same concentration of either drug is vaped alone (Breit et al. 2022). However given that the magnitude of tolerance to hypothermic effects of THC was similar across the THC-only and THC-nicotine groups, and similar to our prior report (Nguyen et al. 2016b), this seems unlikely to have occurred in the present study.

### Volitional nicotine vapor exposure

The rats responded for nicotine in a manner consistent with some criteria for self-administration, as this term is used to describe intentional drug seeking behavior to an individually determined level of intoxication. Session intake under FR1 contingency was relatively consistent from session 2 to session 32 on a group mean basis. Continued drug-taking is a first-principle of self-administration. The declining trends sometimes associated with sequential days of intravenous nicotine self-administration (O'Dell and Koob 2007) were not observed, presumably prevented with the intermittent schedule of sessions. Group means exceeded a 2:1 ratio of drug-associated:non-associated responses, a criterion which has been used to infer self-administration in some studies (Freels et al. 2020; Spencer et al. 2018; Stringfield et al. 2023). Total drug-associated responses increased significantly after the introduction of FR5 and declined again after the restoration of FR1. These are similar to effects previously reported for female rats in a prior study (Gutierrez et al. 2022) and are, together, highly consistent with drug-seeking behavior. This finding supports prior work with different vapor delivery and methodological approaches which also showed nicotine self-administration by vapor inhalation (Cooper et al. 2021; Lallai et al. 2021; Smith et al. 2020).

The present study found that adolescent THC exposure reduces the responding for nicotine vapor in adulthood. This appeared to be a preference set-point, since responding increased under the FR5 contingency and the percentage of drug-associated responses was, if anything, slightly higher than for that of the non-THC-exposed rats. Our prior work shows that the thermoregulatory tolerance produced by repeated inhalation exposure to THC during adolescence (Fig. 1) persists into adulthood (Nguyen et al. 2020b). Thus, the exposure likely produces a persisting downregulation of endogenous cannabinoid receptor 1 (CB_1_) function. Prior work has shown that CB_1_ antagonist and/or inverse agonist compounds acutely inhibit the rewarding effects off nicotine in squirrel monkeys (Schindler et al. 2016) and reduce nicotine self-administration in rats (Cohen et al. 2002; Forget et al. 2009; Shoaib 2008); see Le Foll et alia for review (Le Foll et al. 2008). Conversely, the CB_1_ full agonist WIN 55,212–2 increased responding for nicotine under a PR schedule in squirrel monkeys (Gamaleddin et al. 2012). It is, perhaps, unsurprising that alterations in endocannabinoid function might interact with reinforcing effects of nicotine, since signaling via nicotinic acetylcholine receptors (nAChRs) and endocannabinoid receptors interacts functionally and bidirectionally (Valles and Barrantes 2022).

Interestingly, the addition of menthol to the nicotine vapor did not affect drug seeking behavior (Fig. 8), unlike a prior investigation in mice (Cooper et al. 2021), nor did the increase in nicotine concentration. It is possible that the former was due to the nicotine doses being at the higher end of the dose range and therefore no additional effect of menthol could be detected. The lack of difference when the vapor concentration was increased to 60 mg/mL in the PG is perhaps unexpected but the dose–effect curves for intravenous self-administration of nicotine can be quite flat, e.g., across 2- to threefold differences in dose. For example, female mice self-administered similar intravenous infusions of nicotine whether it was 0.3 or 0.1 mg/kg/infusion (Dukes et al. 2020) and male rats self-administered similar numbers of intravenous infusions of 0.03 or 0.06 mg/kg/infusion nicotine (O’Dell et al. 2007). It is therefore most likely that the 30-mg/mL and 60-mg/mL conditions used here did not produce a large enough effective difference in delivered dose.

The adolescent nicotine inhalation did not affect adult responding for nicotine vapor, whereas adolescent THC exposure reduced nicotine self-administration in this study. This is similar to an effect reported in female mice injected daily during adolescence with nicotine, the CB_1_ full agonist WIN55,212–2 (WIN) or the combination (Dukes et al. 2020), in which adult intravenous nicotine self-administration was lower than the control group in the WIN or WIN + nicotine groups, but unchanged in the nicotine-only group. The group effects were observed at the lowest unit doses of nicotine in a dose-substitution assessment, i.e., at or below the training dose, something that was not assessed in this study.

### Volitional heroin vapor exposure

The difference in volitional responding for nicotine vapor that was associated with adolescent THC exposure appeared to be selective for nicotine. The THC groups obtained more vapor deliveries when heroin was introduced, and this resulted in closing the persisting difference with the no-THC groups. The impact of the volitional heroin vapor exposure on the involuntary anti-nociceptive effect was similar (Fig. 9), further emphasizing the similar level of subjective intoxication. This, combined with the differential effect of adolescent THC versus nicotine vapor exposure on the anti-nociceptive effects of THC, underlines the selectivity of the insult, depending on the drug. Prior studies have shown repeated adolescent injections of THC lead to increased heroin self-administration in acquisition (Ellgren et al. 2007; Lecca et al. 2020), or in yohimbine-induced re-instatement after no difference in acquisition (Stopponi et al. 2014), in adulthood. Adult female rats exposed as adolescents to THC vapor expressed higher rates of self-administration of fentanyl at low unit doses, after no difference in the acquisition of oxycodone self-administration (Nguyen et al. 2020b). Although the primary focus here was on nicotine self-administration, the heroin vapor experiment provides indirect support for prior findings that repeated adolescent THC may lead to a lasting vulnerability to the reinforcing effects of heroin. This interpretation is based on the greater relative change in self-administration behavior for the THC groups vs. the no-THC groups when heroin was substituted for nicotine. A future study initiating self-administration with heroin would be required to provide direct confirmation of this inference.

### Assessing risks across the lifespan

It is notable that these studies successfully assessed drug-seeking behavior into middle age in the rat, with the final heroin vapor self-administration studies completed around 50 weeks of age. Thus, it is possible to use rat models to investigate potential lasting effects of adolescent drug exposure and even age-related factors that may further understanding of, e.g., a recent increase in middle-aged opioid-related fatalities (Monnat 2022). As overviewed in the Introduction, humans express various trajectories of nicotine and cannabis use, and this occur across significant fractions of the lifespan from adolescence into late adulthood. Some of these differences are associated with demographic factors such as race and ethnicity (Boyle et al. 2021; Liu et al. 2023). Controlled animal models are needed to parse factors that may explain differential rates in men versus women, or in ethnic and racial populations, by isolating the effects of drug exposure in the absence of human social factors. This latter is particularly critical given long term NIH funding disparities (Lauer and Roychowdhury 2021; Taffe and Gilpin 2021b) which leave topics of concern to Black PIs, across many neuropsychiatric domains (Gilpin and Taffe 2021; Harnett 2020; Lauer et al. 2021; Taffe 2021) including substance use disorders (Acevedo et al. 2018; CPDD Board 2022; Taffe and Gilpin 2021a), at a significant funding disadvantage (Hoppe et al. 2019). Here, we were able to isolate differential adolescent exposure and then determine lasting consequences throughout the young adult to middle age interval with all subsequent drug exposure similar across these groups. In longitudinal human studies, it would be far more likely that adolescent drug exposure is associated with additional significant differences in drug exposure throughout the early to middle adult age range, complicating inference about the adolescent drug exposure. It should be additionally noted on a practical level that the use of the vapor inhalation model for self-administration overcame typical subject loss due to, e.g., obstructed catheters and catheter-related health problems that might be expected in an intravenous self-administration approach.

Overall, these data do not illustrate significant lasting consequences of repeated adolescent nicotine exposure by vapor inhalation on activity patterns on an exercise wheel, nor on nicotine or heroin vapor self-administration. This was the case when nicotine was administered alone or in the context of coincident THC vapor inhalation. The THC exposure, in contrast, produced lasting consequences as was the case in our prior studies (Nguyen et al. 2018, 2020b). In this case, tolerance to the hypothermic effects of acute THC, lower activity on the running wheel, and reduced nicotine vapor self-administration were observed in adulthood; these effects were not significantly modulated by the addition of nicotine during repeated adolescent exposure.

## Data Availability

Data will be made available to qualified individuals upon legitimate request.
